# Diagnostic value of ^18^F-FDG-PET to predict the tumour immune status defined by tumoural PD-L1 and CD8^+^tumour-infiltrating lymphocytes in oral squamous cell carcinoma

**DOI:** 10.1038/s41416-020-0820-z

**Published:** 2020-04-02

**Authors:** Maria Togo, Takehiko Yokobori, Kimihiro Shimizu, Tadashi Handa, Kyoichi Kaira, Takaaki Sano, Mariko Tsukagoshi, Tetsuya Higuchi, Satoshi Yokoo, Ken Shirabe, Tetsunari Oyama

**Affiliations:** 1 Department of Diagnostic Pathology, Gunma University Graduate School of Medicine, Maebashi, Gunma Japan; 2 Department of Innovative Cancer Immunotherapy, Gunma University, Maebashi, Gunma Japan; 3 Department of General Surgical Science, Graduate School of Medicine, Gunma University, Maebashi, Gunma Japan; 4 Department of Respiratory Medicine, Comprehensive Cancer Center, International Medical Center, Saitama Medical University, Hidaka, Saitama Japan; 5 Department of Diagnostic Radiology and Nuclear Medicine, Gunma University, Maebashi, Gunma Japan; 6 Department of Oral and Maxillofacial Surgery and Plastic Surgery, Gunma University Graduate School of Medicine, Maebashi, Gunma Japan

**Keywords:** Immunosurveillance, Diagnostic markers, Cancer microenvironment

## Abstract

**Background:**

Lately, immune checkpoint proteins, such as programmed death 1 (PD-1) and its ligand-1 (PD-L1), have garnered attention as a new target in oral squamous cell carcinoma (OSCC). Reportedly, fluoro-d-glucose (FDG)-uptake alteration by anti-PD-1 antibody treatment depicts the response in patients with lung cancer. This study aims to elucidate the correlations between tumour immune status, clinicopathological factors, ^18^F-FDG-uptake and cold tumour phenotypes as low PD-L1 expression/low CD8^+^tumour-infiltrating lymphocytes (TILs) in OSCC.

**Methods:**

We performed immunohistochemical analysis of PD-L1, hypoxia-inducible factor 1 A (HIF-1A), glucose transporter type 1 (GLUT1), CD8, E-cadherin and Ki-67 on 59 operable OSCC samples. We assessed the correlations between these factors and preoperative ^18^F-FDG-uptake, clinicopathological characteristics and prognosis.

**Results:**

Low expression of PD-L1 in OSCC correlated with cancer aggressiveness, poor prognosis, high ^18^F-FDG-uptake with HIF-1A/GLUT1 and low E-cadherin expression and low CD8. Cold tumour phenotypes as low PD-L1 tumour cells and low stromal CD8 correlated with the poor prognosis, high ^18^F-FDG-uptake and E-cadherin suppression. Furthermore, the high level of preoperative ^18^F-FDG-uptake in OSCC was an independent predictor of the cold tumour immune status.

**Conclusions:**

^18^F-FDG-uptake is an independent predictor of cold tumour in OSCC. ^18^F-FDG-PET imaging could be a promising diagnostic tool to estimate tumour immune status.

## Background

Although chemotherapy and radiotherapy may confer survival and organ preservation benefits in oral squamous cell carcinoma (OSCC),^1,2^ patients with therapeutic-resistant OSCC exhibit poor prognoses.^3^ Thus, further research is warranted worldwide to recognise new therapeutic targets and enhance the prognosis of patients with resistant OSCC.

Lately, immune checkpoint inhibitors (ICIs) have garnered considerable attention as a new innovative cancer therapy.^4,5^ Studies have demonstrated that targeting immune checkpoint proteins such as programmed death 1 (PD-1) and its ligand-1 (PD-L1) exerts a continuous and significant clinical effect and exhibits low toxicity in some responder patients with various cancer types.^6–10^ Conversely, almost all non-responding patients with cancer have been reported to gain no adequate benefits despite using ICIs.^11^ Thus, presently, several researchers are focusing on the development of useful biomarkers to estimate the sensitivity of ICIs for patients with cancer. In fact, some studies have reported tumour immune status on the basis of PD-L1-positive tumour cells/stromal CD8^+^tumour-infiltrating lymphocytes (TILs), tumour mutation burden and interferon-γ gene signature as promising biomarker candidates for estimating sensitivity to ICIs.^12–14^ Among them, the tumour immune status of tumours with low PD-L1 expression/low activated tumour-specific CD8^+^TILs has been termed ‘cold tumour’, which correlates with poor local immune response and ICIs resistance when compared with the ICIs-sensitive hot tumour with high PD-L1 expression/high CD8^+^TILs.^15,16^ Reportedly, patients with OSCC correlate with suppressed tumour immunoreactivity and high levels of tumour mutation burden, which is associated with a good response to ICIs.^17,18^ Cold tumour has attracted attention as ICIs resistant marker, as well as being one of the therapeutic targets to improve the sensitivity of ICIs; however, the importance of cold tumour in patients with OSCC has not been previously reported. Nevertheless, invasive tumour sampling is essential in order to ascertain tumour immune status, including cold tumour phenotypes, DNA status and gene expression signature in tumour tissues.

Although the use of 2-deoxy-2-[fluorine-18]fluoro-d-glucose with positron emission tomography/computed tomography (^18^F-FDG-PET/CT) has been considered to be a non-invasive diagnostic tool in order to differentiate between cancer tissue and benign lesions, false-positive findings, such as high ^18^F-FDG-uptake, might occur in inflammatory diseases.^19^ Previously, we reported that the change in ^18^F-FDG-uptake before and after anti-PD-1 antibody nivolumab treatment signified the subtle nivolumab response in patients with lung cancer.^20^ These findings indicated the possible correlation between ^18^F-FDG-uptake and the existing ICIs sensitivity markers, such as cold or hot tumour immune status, regarding the PD-L1 and CD8^+^TILs in tumour tissues. In addition, the tumoural PD-L1 expression has been reported to be associated with ^18^F-FDG-uptake in lung and bladder cancers,^20,21^ and hypoxia-inducible factor 1 A (HIF-1A)/glucose transporter type 1 (GLUT1) signalling has been shown to be related to^18^F-FDG-uptake.^22^ Furthermore, there are also reports on the relationship between epithelial–mesenchymal transition (EMT) and ^18^F-FDG-uptake.^23^

However, to date, information regarding the clinicopathological significance, tumour immune status and the non-invasive assessment of ^18^F-FDG-uptake, which was regulated by the accumulation of HIF-1A and GLUT1 and EMT induction characterised by the epithelial marker suppression in OSCC, remains limited. Remarkably, the HIF-1A/GLUT1/EMT axis correlates with not only the cold tumour immune status but also chemoradiation therapy, cancer aggressiveness and poor prognosis.^24–26^

This study aims to elucidate the correlation between tumour immune status, such as PD-L1-positive tumour cells/stromal CD8^+^ TILs, and HIF-1A/GLUT1/EMT expression regarding the ^18^F-FDG-uptake in OSCC.

## Methods

### Patient background

We enrolled patients with OSCC (*n* = 59; 28 males and 31 females) who underwent surgical resection at the Department of Oral and Maxillofacial Surgery, Gunma University Hospital (Maebashi, Gunma, Japan), from January 2009 to March 2014. Of note, no patient underwent chemotherapy or radiotherapy preoperatively. Of 59 patients, 50 received ^18^F-FDG-PET for preoperative assessment. In addition, the tumour staging was based on the Union for International Cancer Control Tumour-Node-Metastasis classification (seventh edition), General Rules for Clinical Studies on Head and Neck Cancer (fifth edition) and the mode of invasion (Y–K classification).^27^ This study conformed to the tenets of the Helsinki Declaration and was approved by the Institutional Review Board for Clinical Research at the Gunma University Hospital (Maebashi, Gunma, Japan; approval number: 2018212). Patients’ agreement was obtained with the opt-out method.

### Immunohistochemistry

We cut a 4-μm section from paraffin blocks of samples, thereafter mounting each section on a silane-coated glass slide, deparaffinising and soaking for 30 min at room temperature in 0.3% H_2_O_2_/methanol to block endogenous peroxidases. Supplementary Table 1 details the staining procedure.

Next, non-specific binding sites were blocked by incubating with 0.25% casein/1% BSA for 30 min at room temperature. The antibodies of PD-L1 (28-8; 1:400 dilution; Abcam, Cambridge, UK), CD8 (C8/144B; 1:100 dilution; DAKO, Glostrup, Denmark), HIF1-A (EP1215Y; 1:200 dilution; Abcam, Cambridge, UK), GLUT1 (ab15309; 1:200 dilution; Abcam, Cambridge, UK), E-cadherin (HECD-1; 1:500 dilution; Takara BIO, Shiga, Japan), and Ki-67 (MIB-1; 1:40 dilution; DAKO, Glostrup, Denmark) were visualised using the HRP/DAB (Polymer) Kit (Nichirei, Tokyo, Japan) and Histofine Simple Stain MAX-PO (Multi) Kit (Nichirei) per the manufacturer’s instructions. We applied the chromogen 3,3-diaminobenzidine tetrahydrochloride (Dojindo Laboratories, Kumamoto, Japan) as a 0.02% solution containing 0.005% H_2_O_2_ in 50-mM Tris–HCl buffer (pH 7.6). Thereafter, the sections were lightly counterstained with Mayer’s haematoxylin and mounted. Finally, we established negative controls by omitting the primary antibody.

### Assessment of immunohistochemistry, TILs and ^18^F-FDG-uptake

We evaluated tumour cells showing membranous staining for PD-L1 as positive cells. The following semi-quantitative scoring method was used for PD-L1: 1, <1%; 2, 1–5%; 3, 6–10%; 4, 11–25%; 5, 26–50% and 6, >50% of positive cells. Notably, tumours with a score ≥3 were graded as high expression.^28^ In addition, we used haematoxylin and eosin slides to assess the infiltration of TILs in the tumour stroma as recommended by the International TILs Working Group.^29^ We assessed the percentages of the CD8 expression when compared with the total amount of nucleated cells in the stromal compartments on the basis of previous studies.^30^ We used the median values as a cut-off point to establish bicategorical parameters (low vs high) for TILs count and CD8 expression. The median TILs and CD8 were 30% (range, 10–80%) and 20% (range, 0–60%), respectively. We considered tumour cells expressing membranous GLUT1 and nuclear HIF-1A as positive cells; they were evaluated in five fields (×400) to determine the proportion of positive cells.^22^ For GLUT1 and HIF-1A, we used a semi-quantitative scoring method as follows: 1, < 10%; 2, 10–25%; 3, 25–50%; 4, 51–75% and 5, >75% of cells positive. We determined the positive cell score ≥3 as high expression of GLUT1 and HIF-1A. Moreover, E-cadherin was stained on the cell membrane as positive, for which the following scoring used a 4-point scale:^31^ 0, complete absence or negative; 1, <10% bright membrane expression; 2, ≥10% but ≤50% membrane expression; and 3 = >50% membrane expression. Of note, E-cadherin-positive cell score ≥2 was considered to be a positive expression. For Ki-67, we evaluated a highly cellular area of the immunostained sections. We defined all epithelial cells with nuclear staining of any intensity as high expression. Approximately 1000 nuclei were counted on each slide. The proliferative activity was assessed as the percentage of Ki-67–stained nuclei (Ki-67 labelling index) in the sample; the median value of the Ki-67 labelling index was 22% (range, 2–69%). Furthermore, at least two authors who were blinded to the data assessed the sections using a light microscope (Olympus Corporation, Tokyo, Japan).

**Fig. 1:**
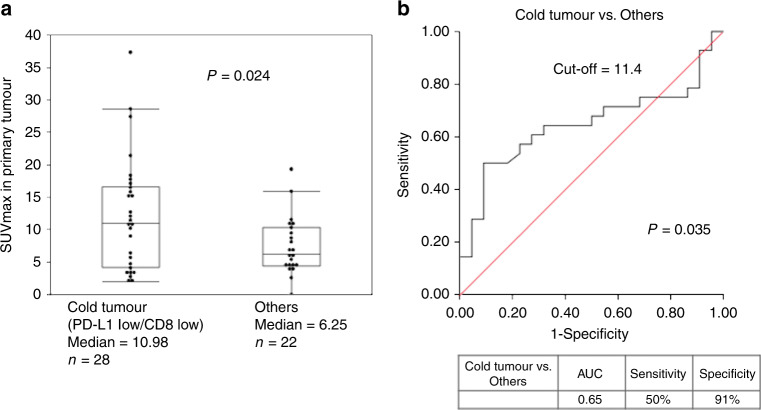
The median ^18^F-FGD-uptake of a primary tumour was 8.85 (range: 0–37.3). ^18^F-FDG-uptake was compared between the cold tumour group and the others group. In the cold tumour group, median ^18^F-FDG-uptake was 10.98 while it was 6.25 in the others group. **a** shows independent samples median test (*P* = 0.024). ROC analysis showed that the optimal cut-off value for a cold tumour was 11.4 (AUC = 0.65, *P* = 0.04, sensitivity = 50%, specificity = 91%; **b** Patients with ^18^F-FDG-uptake above 11.4 for cold tumours were defined as having high ^18^F-FDG-uptake.

In this study, the median ^18^F-FGD-uptake of a primary tumour was 8.85 (range, 0–37.3). ^18^F-FDG-uptake was compared between the cold tumour group and the others group. The median ^18^F-FDG-uptake of the cold tumour group was 10.98, whereas it was 6.25 in the others group. Figure 1a shows independent samples median test (*P* = 0.024). The receiver operating characteristic curve (ROC) analysis showed that the optimal cut-off value for a cold tumour was 11.4 [area under the curve (AUC), 0.65; *P* = 0.04, sensitivity, 50%, specificity, 91%; Fig. 1b]. Patients with ^18^F-FDG-uptake ≥11.4 for a cold tumour were defined as having high ^18^F-FDG-uptake.

### PET imaging

All the patients fasted for at least 6 h before PET imaging, which was performed using a PET/CT scanner (Discovery STE; GE Healthcare, USA) with a 700-mm field of view at the Gunma University Hospital. Thereafter, we imitated three-dimensional data acquisition 50 min after intravenously injecting 5 MBq/kg of ^18^F-FDG. On the basis of the range of imaging, we acquired 4–10 bed positions (3-min acquisition per bed position). In addition, attenuation-corrected transverse images obtained with ^18^F-FDG were reconstructed with the ordered-subsets expectation maximisation algorithm into 128 × 128 matrices with a 3.27-mm slice thickness. Two experienced nuclear physicians who were blinded to patients’ clinical history and data interpreted all ^18^F-FDG images. Any discrepant results between the physicians were resolved by consensus. Furthermore, functional images of the standardised uptake value (SUV) were produced using attenuation-corrected transaxial images, the injected doses of ^18^F-FDG, the patients’ body weight and the cross-calibration factor between PET and the dose calibrator. We defined the SUV as follows: SUV = radioactive concentration in the region of interest (ROI) [MBq/g]/injected dose (MBq)/patient’s body weight (g). Of note, the ROI was manually drawn over a primary tumour on the SUV images. When a tumour was >1 cm in diameter or its shape was irregular or multifocal, an ROI of ~1 cm in diameter was drawn over the area corresponding to the maximal tracer uptake. A nuclear physician performed the ROI analysis using corresponding CT scans. In this study, we used the maximal SUV (SUV_max_) in the ROI as a representative value for the assessment of ^18^F-FDG-uptake in the primary lesion. Furthermore, we performed CT scanning for initial staging with an intravenous contrast medium, and board-certified radiologists interpreted the CT images.

### Statistical analysis

In this study, statistical analyses were performed using the Mann–Whitney U-test for continuous variables, whereas the χ^2^ test and analysis of variance (ANOVA) were used for categorical variables. In addition, the Kaplan–Meier method was used to create survival curves. We used the log-rank test to assess the differences between survival curves. In addition, univariate and multivariate analyses were performed with each predictive factor using logistic regression analysis. AUC of ROC, sensitivity and specificity were calculated to detect the optimal cut-off values for ^18^F-FDG-uptake parameters of cold tumours. We considered *P* < 0.05 to be statistically significant. JMP software version 14 (SAS Institute, Cary, NC, USA) was used to perform all statistical analyses.

## Results

### Correlation between the PD-L1 expression and clinicopathological characteristics of OSCC

**Fig. 2:**
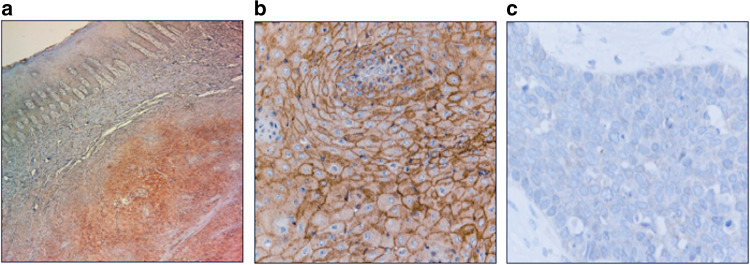
The immunohistochemical analysis of the programmed death ligand-1 (PD-L1) expression in representative oral squamous cell carcinoma (OSCC) samples. **a** high PD-L1 expression in a representative OSCC tissue compared with adjacent normal oral epithelial tissue (40× magnification). **b** the high-power view of high PD-L1 expression in a representative OSCC tissue (×200 magnification). **c** the high-power view of low PD-L1 expression in a representative OSCC tissue (×200 magnification).

**Fig. 3:**
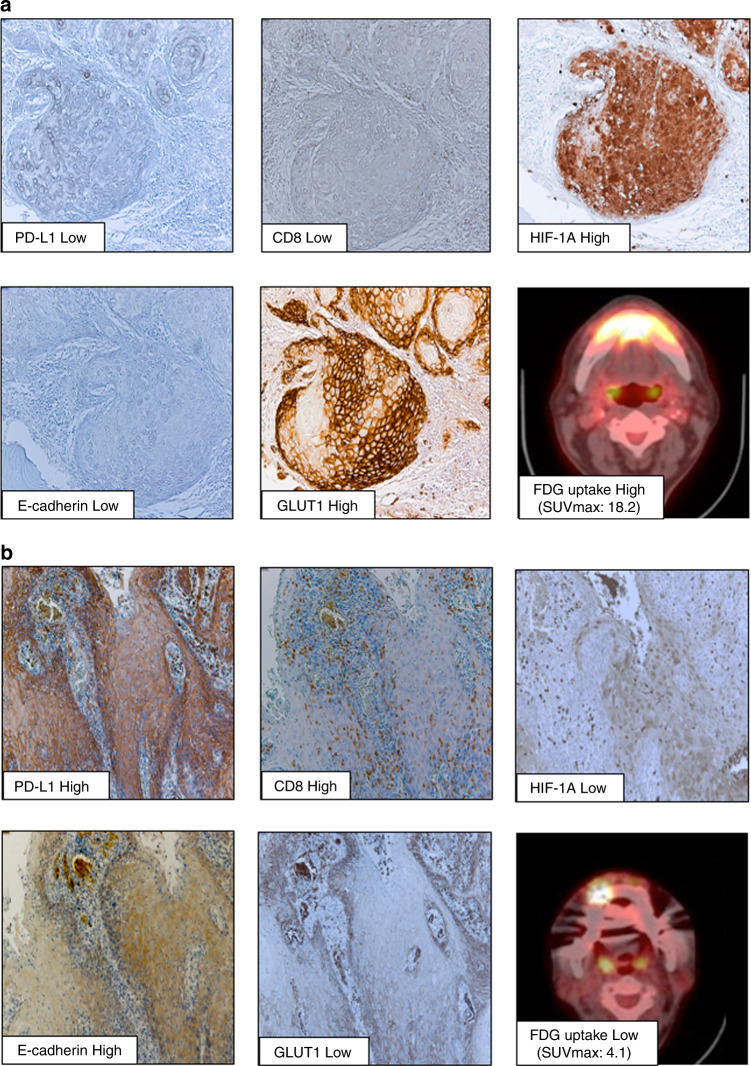
Fluoro-d-glucose with positron emission tomography (FDG-PET) imaging and immunohistochemical analysis of programmed death ligand-1 (PD-L1), CD8, hypoxia-inducible factor 1 subunit alpha (HIF-1A), E-cadherin, and glucose transporter type 1 (GLUT1) in oral squamous cell carcinoma (OSCC) tissues from representative identical patients. **a** the OSCC tissue with high ^18^F-FDG-uptake shows low expression of PD-L1 and E-cadherin, high expression of HIF-1A and GLUT1 in tumour cells and low stromal CD8, suggesting a representative cold tumour tissue (×200 magnification). **b** the OSCC tissue with low ^18^F-FDG-uptake shows high expression of PD-L1 and E-cadherin, low expression of HIF-1A and GLUT1 in tumour cells, and high tumour stromal CD8^+^TILs, suggesting a representative hot tumour tissue (×200 magnification).

We assessed the PD-L1 expression using immunohistochemistry (IHC) in 59 OSCC samples; PD-L1 immunostaining was predominantly localised in the plasma membrane of OSCC. The PD-L1 expression levels in tumour samples were higher than those in normal samples (Fig. 2a); the representative high PD-L1 expression is shown in Fig. 2b and the representative low expression is shown in Fig. 2c. Of 59 OSCC specimens, 38 (64.4%) were assigned to the low PD-L1 expression group as PD-L1 score 1 and 2 (Fig. 3a) and 21 (35.6%) to the high PD-L1 expression group as score ≥3 (Fig. 3b). The percentage of PD-L1 levels with scores of 1, 2, 3, 4, 5 and 6 were 20% (12/59), 44% (26/59), 6% (4/59), 10% (6/59), 6% (4/59) and 12% (7/59), respectively. The percentages of CD8^+^TILs expression were compared with the total amount of nucleated cells in the stromal compartments. CD8^+^TILs-positive cell score ≥20% was considered to be positive expression. The positive expression of GLUT1 and E-cadherin was predominantly localised in the membrane, whereas that of HIF-1A and Ki-67 was localised in the nuclei. The rate of high CD8 + TILs, GLUT1 and HIF-1A was recognised in 42% (25/59), 47% (28/59) and 58% (34/59) patients, respectively. E-cadherin stained on the cell membrane was identified in 29% (17/59) patients as positive. Ki-67 was stained in the nuclei; the median value of the Ki-67 labelling index was 22% (range, 2–69%).

Table 1 presents the correlations between PD-L1 expression in OSCC specimens and clinicopathological characteristics of patients: the low PD-L1 expression group markedly correlated with the Y–K classification; tumour infiltration; high ^18^F-FDG-uptake; high expression of HIF-1A/GLUT1; low levels of CD8, TILs and Ki-67 and negative expression of E-cadherin (Fig. 3a) compared with those of the high PD-L1 expression group (Fig. 3b; Table 1).

**Table 1:** The relationship of clinicopathological factors, PD-L1, cold tumour phenotype, and FDG-uptake in clinical OSCC patients.

Factors	PD-L1 expression			Cold tumour^a^ vs others			FDG-uptake		
	Low	High	*P-*value	Cold tumours	Others	*P*-value	Low	High	*P*-value
	*n* = 38	*n* = 21		*n* = 34	*n* = 25		*n* = 27	*n* = 23	
Age									
	69.3 ± 14.7	72.7 ± 12.2	0.321	70.3 ± 15.1	70.9 ± 12.4	0.982	72 ± 13.8	72.3 ± 9.6	0.661
Gender									
Male	21	7	0.106	19	9	0.131	12	12	0.586
Female	17	14		15	16		15	11	
Location									
Tongue	10	5	0.438	10	5	0.301	9	1	0.046
Gingiva	23	12		21	14		13	19	
Buccal mucosa	3	2		2	3		2	2	
Oral floor	1	2		1	2		2	1	
Lip	1	0		0	1		1	0	
Tumour size									
<2 cm	10	6	0.852	10	6	0.644	10	1	0.005*
≧2 cm	28	15		24	19		17	22	
Differentiation									
Well	24	15	0.305	22	17	0.767	16	18	0.304
Moderately	10	6		9	7		10	4	
Poorly	4	0		3	1		1	1	
T factor									
T1	11	6	0.885	11	6	0.598	10	1	0.018*
T2	14	7		11	10		9	10	
T3	6	5		5	6		6	5	
T4	7	3		7	3		2	7	
N factor									
Negative	33	20	0.307	30	23	0.636	25	19	0.279
Positive	5	1		4	2		2	4	
Metastasis									
Negative	38	21	0	34	25	0	27	23	0
Positive	0	0		0	0		0	0	
Lymphatic invasion									
Negative	22	17	0.073	20	19	0.168	24	8	<0.001*
Positive	16	4		14	6		3	15	
Venous invasion									
Negative	27	19	0.085	25	21	0.338	25	14	0.007*
Positive	11	2		9	4		2	9	
Y–K classification									
4CD	20	4	0.012*	18	6	0.025*	10	9	0.879
1, 2, 3	18	17		16	19		17	14	
INF									
a	3	6	0.018*	3	6	0.056	4	3	0.979
b	15	11		13	13		13	11	
c	20	4		18	6		10	9	
Stage									
I	11	6	0.448	11	6	0.151	10	1	0.004*
II	12	6		11	7		8	8	
III	5	6		3	8		7	4	
IV	10	3		9	4		2	10	
FDG-uptake									
Low	12	15	0.003*	14	20	0.002*	–		
High	20	3		14	2				
HIF1-A									
Low	11	14	0.005*	7	6	0.755	16	2	0.0002*
High	27	7		27	19		11	21	
GLUT1									
Low	14	17	0.001*	11	20	0.0003*	21	5	<0.0001*
High	24	4		23	5		6	18	
CD8									
Low	34	0	<0.0001*	–			11	17	0.019*
High	4	21					16	6	
TILs									
Low	24	7	0.028*	24	7	0.0007*	13	11	0.981
High	14	14		10	18		14	12	
E-cadherin									
Negative	33	9	0.0004*	30	12	0.007*	15	19	0.041*
Positive	5	12		4	13		12	4	
Ki-67 labeling index									
	18.25 ± 14.9	29.3 ± 12.6	0.0018*	19.1 ± 15.4	26.4 ± 13.6	0.0241*	23.6 ± 14	20 ± 14.1	0.419

*Well* well differentiated, *Moderately* moderately differentiated, *Pooly* poorly differentiated, *Y–K* Yamamoto-Kohama classification, *4CD* grade4C and grade4D, *INF* Infiltrative pattern.

*p  <  0.05 is considered statistically significant.

^a^Cold tumour, low tumoural PD-L1 and low intratumoural CD8+ T cells.

In this study, we defined OSCC samples with low tumoural PD-L1 and low stromal CD8^+^TILs as cold tumours, which reportedly correlates with the low anti-tumour immune reactivity.^15,16^ Patients with a cold tumour markedly correlated with the Y–K classification, high ^18^F-FDG-uptake, high expression of GLUT1, low levels of TILs and negative expression of E-cadherin when compared with those of others (Table 1). Moreover, the patients with high FDG-uptake were significantly associated with the progression of tumour size, T factor, lymphatic invasion, venous invasion, stage, the high expression of HIF1-A and GLUT1 and the low expression of CD8, PD-L1 and E-cadherin compared to those with low FDG-uptake (Table 1).

### Prognostic significance of the PD-L1 expression and cold tumour phenotype in patients with OSCC

**Fig. 4:**
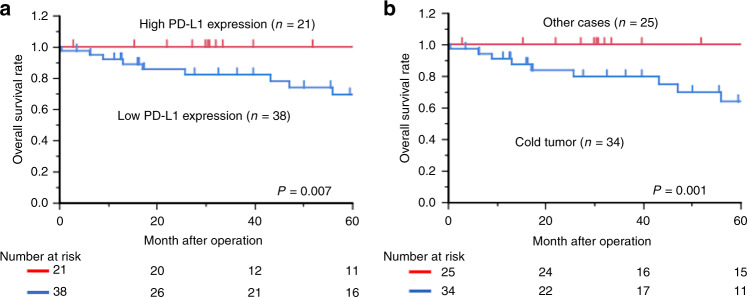
Kaplan–Meier curves based on the tumour programmed death ligand-1 (PD-L1) expression and stromal CD8^+^tumour-infiltrating lymphocytes (CD8^+^TILs) in clinical oral squamous cell carcinoma (OSCC) samples. **a** the overall survival curves according to the PD-L1 expression in patients with OSCC (*n* = 59; *P* = 0.007). **b** the overall survival curves according to the cold tumour immune status as low tumour PD-L1 expression and stromal CD8^+^TILs in patients with OSCC (*n* = 59; *P* = 0.001).

Figure 4 presents the prognostic significance of PD-L1 expression and cold tumour phenotype on the overall survival. The overall survival rates of the low PD-L1 expression group (*n* = 38) were markedly worse than those of the high expression group (*n* = 21; *P* = 0.007; Fig. 4a). Furthermore, the overall survival rates of patients with OSCC with a cold tumour (*n* = 34) were markedly worse than those of other cases (*n* = 25; *P* = 0.001; Fig. 4b).

### Diagnostic value of the ^18^F-FDG-uptake to estimate the cold tumour immune status in OSCC

In this study, we analysed the diagnostic value of clinicopathological factors for predicting the cold tumour immune status in OSCC because of the high prognostic relevance of cold tumours in patients with OSCC. Remarkably, the multivariate analysis revealed that the assessment of high ^18^F-FDG-uptake by non-invasive ^18^F-FDG-PET was an independent predictive factor for the cold tumour immune status (OR, 29.17; 95% CI, 2.79–304.2; *P* = 0.005; Table 2).

**Table 2:** Univeriate and multivariate analyses of the predictive factors related to evaluation of cold tumour immune status.

Factors	Univariate analysis			Multivariate analysis		
	OR	95% CI	*P*-value	OR	95% CI	*P-*value
Age (<73/≧73) (yr.)	0.65	0.22–1.82	0.41	–	–	–
Gender (male/female)	2.25	0.78–6.50	0.13	4.71	1.18–18.7	0.03
Tumour size (<2 cm/≧2 cm)	1.31	0.4–4.28	0.65	–	–	–
T factor (T1, 2/T3, 4)	1.03	0.35–3.03	0.96	–	–	–
N factor (negative/positive)	0.65	0.11–3.88	0.64	–	–	–
Lymphatic invasion (negative/positive)	0.45	0.14–1.42	0.17	1.31	0.17–9.93	0.79
Venous invasion (negative/positive)	0.53	0.14–1.97	0.34	2.18	0.21–22.1	0.51
Y–K classification (1, 2, 3/4 C, 4D)	3.56	1.14–11.12	0.029	7.39	1.57–34.7	0.01
Levels of FDG-uptake (Low/High)	10	1.96–51.1	0.006	29.17	2.79–304.2	0.005

*OR* odds ratio, *CI* confidence interval.

## Discussion

This study elucidated that the low PD-LI expression in OSCC correlated with cancer aggressiveness, poor prognosis, high ^18^F-FDG-uptake with HIF-1A/GLUT1 expression, low E-cadherin expression and low CD8^+^TILs. In addition, cold tumour phenotypes as low PD-L1 tumour cells and low stromal CD8^+^TILs correlated with poor prognosis, high ^18^F-FDG-uptake and E-cadherin suppression. Remarkably, the assessment of high ^18^F-FDG-uptake using non-invasive ^18^F-FDG-PET scanning was an independent predictor of the cold tumour phenotype in OSCC.

Reportedly, the expression of PD-L1, which belongs to the B7/CD28 superfamily, is higher in cancer tissues than it is in non-cancerous tissues^32^ and cancer tissues with high PD-L1 expression correlate with cancer aggressiveness and poor prognosis.^33,34^ Conversely, some other studies have reported the opposite correlation between prognosis and PD-L1 expression in cancer tissues, including OSCC and lung SCC,^35^ highlighting the debatable expression significance of PD-L1 in SCC tissues. This study validated the prognostic significance of low PD-L1 expression in OSCC as a poor prognostic factor. In addition, low PD-L1 expression in tumour cells correlated with high HIF-1A expression and low E-cadherin expression. Polyak and Weinberg reported that the suppression of the epithelial marker, E-cadherin, is a typical characteristic of EMT induction, which is a crucial regulatory mechanism of invasion and metastasis in cancer.^36^ Remarkably, the HIF-1A accumulation causes EMT by upregulating ZEB1 and Wnt/β-catenin,^37,38^ and EMT induction suppresses PD-L1 expression.^39^ These studies indicated that OSCC patients with low PD-L1 expression could have a poorer prognosis than those with high PD-L1 expression through HIF-1A–induced EMT.

Lately, the tumour immune status, such as cold or hot tumours, has garnered substantial attention as a predictive biomarker and therapeutic target to enhance the ICI sensitivity.^40^ Hot tumours with high PD-L1 expression and high CD8^+^TILs correlate with the high sensitivity of immune checkpoint blockades. Contrarily, cold tumours with low PD-L1 expression and low CD8^+^TILs correlate with their therapeutic resistance. Reportedly, HIF-1A–induced GLUT1 overexpression causes the accumulation of lactic acids in the tumour microenvironment, resulting in the suppression of CD8^+^TILs. Besides, PD-L1 expression is suppressed by the activation of the HIF-1A–EMT axis as mentioned above. Remarkably, both GLUT1 overexpression and EMT induction correlate with high ^18^F-FDG-uptake.^41^

In this study, we validated the positive correlations between high.

^18^F-FDG-uptake, high HIF-1A/GLUT1/EMT and cold tumour immune status in patients with OSCC. The high expression levels of PD-L1 in cancer tissues has been previously reported to be related to high ^18^F-FDG-uptake in several cancer patients, contrary to our OSCC data.^20,21^ As mentioned above, it has been reported that the PD-L1 expression is positively regulated by HIF-1A activation and that HIF-1A-induced GLUT1 expression is related to high PD-L1 expression and EMT induction, which is characterised by E-cadherin suppression and cancer aggressiveness.^42,43^ Conversely, PD-L1 expression has been reported to be down-regulated by EMT induction, and the high ^18^F-FDG-uptake in cancer tissues is negatively related to low E-cadherin expression as one of the EMT phenotypes.^23^ Interestingly, the HIF-1A/GLUT1/EMT axis has been known to regulate PD-L1 expression as well as ^18^F-FDG-uptake;^22,28^ however, the relationship between PD-L1 expression, FDG-uptake, and the HIF-1A/GLUT1/EMT axis in identical OSCC samples has not yet been investigated. In this study, we could clarify the significant opposite correlation of high ^18^F-FDG-uptake and low PD-L1 expression with respect to the activation of the HIF-1A/GLUT1/EMT axis in patients with OSCC for the first time. Although the underlying mechanism regarding PD-L1 regulation in OSCC remains unclear to date, the tumoural PD-L1 may be suppressed by the activation of the HIF-1A/GLUT1/EMT axis with regard to the high ^18^F-FDG-uptake in OSCC, this is in contrast to the PD-L1 regulation in other cancers, which are induced by the activation of the HIF-1A signal.^44^ Thus, these findings suggest that high ^18^F-FDG-uptake in OSCC may be associated with low PD-L1 expression because of the difference in PD-L1 regulation mechanisms between OSCC and other cancers. Although the underlying mechanism regarding the hot or cold tumour immune status remains unclear to date, we considered OSCC tumours with high ^18^F-FDG-uptake to have cold tumour characteristics as low PD-L1 and low CD8^+^TILs partially by the activation of the HIF-1A/GLUT1/EMT axis.

Recent research has focused on the tumour mutation burden, interferon-γ gene signature and tumour immune status on the basis of PD-L1 expression and CD8^+^TILs as representative predictive candidates of immunotherapy.^14,37,45^ However, invasive tumour tissue sampling is warranted to analyse the protein expression and extract the DNA and RNA derived from tumour tissues. In this study, we used IHC of resected OSCC samples to assess the relationship between FDG-uptake and the tumour immune status, such as cold or hot tumour, and established that high ^18^F-FDG-uptake by non-invasive ^18^FDG-PET scanning markedly correlated with the progression of clinicopathological factors and cold tumour immune status regarding immunotherapy resistance. The focus now must be on understanding the genetic signatures most likely to be associated with a productive response to ICI therapy.^46^ Thus, our data suggested that non-invasive assessment of ^18^F-FDG-uptake potentially identifies the microenvironment of tumours with low CD8 + TILs and low expression of PD-L1 and that the evaluation of ^18^F-FDG-uptake has a potential to become a crucial biomarker for determining the cancer aggressiveness and adaptation of ICI treatment in patients with OSCC in the future.

This study had some limitations. First, this was a retrospective single-institution study, and we enrolled only patients without ICIs treatment with anti-PD-1 antibodies, such as nivolumab or pembrolizumab, who had undergone surgical resection, creating an unavoidable bias. Second, the number of patients in the study was small. Recently, patients with OSCC often receive the neo-adjuvant therapy to reduce the tumour size before surgery because surgical resection of large primary tumours without neo-adjuvant therapy may cause severe and miserable cosmetic complications. Therefore, it was difficult to increase the samples number of patients with OSCC without neo-adjuvant therapy in this retrospective observational study despite our institution being identified as one of the high-volume university hospitals in Japan. Third, the relationship between tumoural FDG-uptake and systemic inflammatory response is not examined in this study; however, Dolan RD et al., have reported that such systemic inflammatory responses influence the FDG-uptake in tumour tissues.^47^ Therefore, we should analyse the relation of FDG-uptake and local tumour immune status in patients with OSCC with or without systemic inflammatory response in future studies. Finally, we did not implement any functional studies on the associations between low PD-L1, low CD8 + TILs, ^18^F-FDG-uptake and HIF-1A/GLUT1/EMT axis.

In conclusion, this study establishes that low PD-L1 expression in OSCC correlates with cancer progression and poor prognosis. The assessment of the PD-L1 expression level could be a biomarker of aggressive phenotypes and poor survival in patients with OSCC. In addition, high levels of ^18^F-FDG-uptake in OSCC markedly correlate with low PD-L1 expression and low CD8^+^TILs, suggesting the cold tumour phenotype. Overall, this study suggests that the non-invasive assessment of ^18^F-FDG-uptake using ^18^F-FDG-PET could be a promising diagnostic tool to identify cold tumours.

## Supplementary information

Supplementary table 1 - REVISED

## Data Availability

All data generated or analysed during this study are included in this published article and its supplementary information data.
