# Supplementary material for: Diagnostic value of 18F-FDG-PET to predict the tumour immune status defined by tumoural PD-L1 and CD8+tumour-infiltrating lymphocytes in oral squamous cell carcinoma
Source: Br J Cancer. 2020 Apr 2;122(11):1686–94. doi: 10.1038/s41416-020-0820-z (PMC7250916; doi:10.1038/s41416-020-0820-z)
Supplement: Supplementary file 1 — Supplementary table 1 - REVISED [file 41416_2020_820_MOESM1_ESM.docx]

| Supplementary table1. Experimental condition of immunohistochemistry in this study | | | | |
| --- | --- | --- | --- | --- |
| Antibody | Clone | Vender | Dilution | Antigen retrieval |
| PD-L1 | 28-8 | abcam | 1:400 | autoclave in 10mM citrate buffer (pH 6.1) 121℃ 10min |
| CD8 | C8/144B | Dako | 1:100 | boiled 10 mM citrate buffer (pH 6.0) at 98°C for 30 min. |
| HIF1-A | EP1215Y | abcam | 1:200 | heated in boiled water and Immunosaver solution (Nisshin EM, Tokyo, Japan) at 98°C for 30 min |
| GLUT1 | ab15309 | abcam | 1:200 | heated in boiled water and Immunosaver solution (Nisshin EM, Tokyo, Japan) at 98°C for 30 min |
| E-cadherin | HECD-1 | TaKaRa | 1:500 | boiled 10 mM citrate buffer (pH 6.0) at 98°C for 30 min. |
| Ki-67 | MIB-1 | Dako | 1:40 | boiled 10 mM citrate buffer (pH 6.0) at 98°C for 30 min. |
